# Serological profiling of rabies antibodies by enzyme-linked immunosorbent assay and its comparative analysis with rapid fluorescent focus inhibition test in mouse model

**DOI:** 10.14202/vetworld.2019.126-130

**Published:** 2019-01-23

**Authors:** Ashis Debnath, Dinesh C. Pathak, Narayan Ramamurthy, Gulam Mohd, A. B. Pandey, Vikramaditya Upmanyu, A. K. Tiwari, R. Saravanan, Madhan Mohan Chellappa, Sohini Dey

**Affiliations:** 1 Recombinant DNA Laboratory, Division of Veterinary Biotechnology, Indian Veterinary Research Institute, Izatnagar, Bareilly, Uttar Pradesh, India; 2 Division of Biological Standardization, Indian Veterinary Research Institute, Izatnagar, Bareilly, Uttar Pradesh, India; 3 Immunology Section, Indian Veterinary Research Institute, Izatnagar, Bareilly, Uttar Pradesh, India

**Keywords:** comparison, enzyme-linked immunosorbent assay, inactivated vaccine, rabies, rapid fluorescent focus inhibition test

## Abstract

**Aim:**

In this study, we have used enzyme-linked immunosorbent assay (ELISA) as an alternative test to replace the cumbersome rapid fluorescent focus inhibition test (RFFIT) to ascertain the immune status of immunized mice against rabies virus.

**Materials and Methods:**

Rabies is a devastating disease worldwide caused by rabies virus. Proper usage of pre- or post-exposure rabies vaccine can prevent the disease transmission. In this study, mice were immunized with Vero cell-adapted inactivated rabies vaccine. RFFIT was used as a test to determine the serum neutralizing titers in infected/vaccinated mice. Seroprofiling of mice sera was done *in vitro* by ELISA.

**Results:**

Twenty-one days post-immunization, both ELISA and RFFIT assays indicated similar antibody levels in mice sera that were immunized with Vero cell-adapted inactivated rabies vaccine. Both the tests were correlated, and the linearity was verified by the regression line (R²=0.979).

**Conclusion:**

In this study, we profiled the serological status of Vero cell-adapted inactivated rabies vaccine through ELISA in mice model that correlated well with the OIE gold standard test RFFIT.

## Introduction

Rabies is fatal zoonotic viral disease. Rabies virus is a neurotropic, negative sense, single-stranded RNA virus that belongs to the family Rhabdoviridae, genus *Lyssavirus* [1]. The causative virus can spread from saliva of infected animals to others through neuromuscular route [2]. It has been reported that around 55,000 human deaths occur due to rabies infection out of the millions exposed each year worldwide [3]. The disease is endemic in nature and poses a risk to international travelers, particularly, in Asian countries [4-9]. The only way to prevent this disease is to get proper vaccination using pre- or post-exposure vaccines [10]. The potency of an inactivated rabies vaccine is assessed by measuring the serum neutralizing titers in the vaccinated mice by rapid fluorescent focus inhibition test (RFFIT) assay. The antibody titers determine the potency requirement of the rabies vaccines [11].

The RFFIT is considered as the gold standard test for assessing the viral neutralizing antibodies against rabies virus [12]. Nowadays, enzyme-linked immunosorbent assay (ELISA) is being used as an alternative test to RFFIT to detect the rabies antibodies in human sera samples [13,14]. In this study, an indirect ELISA assay based on the whole virus as an antigen was developed. The test developed could be used as an alternative to RFFIT which is difficult to perform at weekly intervals. The antigen used for developing ELISA is from a vaccine strain so safe to handle and does not require BSL3 facilities, whereas, in other studies, either of monoclonal antibodies was used to develop a competitive ELISA or purified glycoprotein antigen has been used. The same vaccine strain was injected into the mice, and hence, the antibodies generated against the whole virus could be easily detected at weekly intervals by the in-house developed kit. Despite being an antigen-antibody binding assay, ELISA has been chosen as it is simple, easy to perform, less time-consuming and does not need a virology laboratory [3,15].

In this study, an ELISA test was standardized and compared with RFFIT at 21 days post-immunization (dpi) mouse sera.

## Materials and Methods

### Ethical approval

In this study, the care and use of the mice were followed according to the Institutional Animal Ethics Committee (IAEC) guidelines (IAEC number is F.26-1/2015-16/J.D (R)/part file, dated October 16^th^, 2017).

### Animals

Three-week-old female Swiss albino mice used in this study were obtained from Laboratory Animal Resource Facility, Indian Veterinary Research Institute (IVRI), Izatnagar.

### Challenge virus and cells

The rabies virus CVS-11 strain was used as a challenge virus and maintained at Division of Biological Standardization, IVRI, Izatnagar. Purified Vero cell-derived inactivated rabies vaccine was procured from Sun Pharmaceutical India Ltd. BHK-21 cells were maintained at Recombinant DNA Laboratory, Division of Veterinary Biotechnology, IVRI, Izatnagar.

### Immunization schedule in mice

Mice were housed 1 week before the primary immunization, and the blood was collected randomly from them. The control unvaccinated mice group (n=6) was injected with PBS. 4-week-old mice (n=6) were primarily immunized with 100 µl (>0.5 IU) of rabies vaccine through intramuscular route in the thigh muscle. A booster dose (>0.5 IU) was given 14 days later. Blood was collected from retro-orbital sinus of vaccinated and unvaccinated mice (after primary immunization) at weekly intervals on 7 dpi, 14 dpi, and 21 dpi. Sera were separated from blood and kept at −20°C for further use.

### ELISA procedure

#### Whole virus antigen coating

Purified Vero cell-derived inactivated rabies vaccine (>2.5 IU) was reconstituted as per the manufacturer’s instructions. The vaccine was initially diluted at 1:50 (V/V) in coating buffer (100 mM bicarbonate buffer, pH 9.6) followed by serial dilution in a flat-bottom polystyrene microtiter 96-well ELISA plate (Greiner Bio-One, Monroe, USA) and incubated overnight at 4°C. By checkerboard titration, the optimal antigen concentration was determined in a definite volume of diluents [9]. Plates were then incubated with blocking solution (PBS with 2% (W/V) bovine serum albumin) for 1 h at 37°C. The plates were washed 3 times with PBS supplemented with 0.05% Tween 20 (PBST) and stored at 4°C until use.

### Test sera

A total of six negative and six positive sera samples were confirmed by RFFIT and diluted in PBS just before use. The optimal dilution of sera was determined by checkerboard titration.

### Peroxidase conjugate

A volume of 100 μl of sheep anti-mouse IgG peroxidase conjugate (Sigma, St. Louis, USA) diluted 1:3000 (V/V) was added to each test and to the control wells. The optimal concentration of anti-mouse IgG peroxidase conjugate was determined by checkerboard titration.

### ELISA procedure

The levels of antibody in the experimental mice were quantified by ELISA at 7, 14, and 21 dpi. Sera were added to the previously antigen-coated plate and incubated at 37°C for 1 h. Following the incubation period, plates were washed 3 times with PBST and incubated with HRP conjugated anti-mouse secondary antibody 1:3000 (V/V) (Sigma, USA) at 37°C for 1 h. Following the incubation period, the plates were washed as described above and finally developed with the substrate solution (100 mM citrate-phosphate buffer containing 1 mg/ml O-phenylenediamine and 1 µl/ml of 30% of H_2_O_2_). Finally, after 15 min, the reaction was stopped by adding 50 µl of 8N H_2_SO_4_, and the absorbance was measured at 490 nm in ELISA reader (Bio-Rad, USA) [16].

The level of anti-rabies antibody was expressed as mean absorbance at optical density 490 nm (OD _490_ nm). To determine the cutoff, the OD values of the sera from the control mice were taken, and the mean OD value was calculated. The mean OD value + 3 standard deviation (SD) was considered as the cutoff value, above which the sera samples were considered as positive.

### RFFIT

RFFIT was performed as per the standard protocol [17]. Blood was collected from each mouse from different groups. Briefly, 50% endpoint titer of the challenge virus CVS-11 was determined as 100 focus-forming dose 50. Challenge rabies virus was added to each chamber. 50 µl of serial 1:5, 1:25, 1:125, 1:625, and 1:3125 dilutions of serum were prepared in Lab-Tek Chamber Slides (Nalge Nunc International, Rochester, NY). The slides were then incubated for 90 min at 37°C. Healthy freshly prepared BHK-21 (5×10^5^ cells) cells were added into each chamber uniformly, and the slide was incubated at 37°C for 23 h. After getting uniform cell monolayer, the slide was washed gently 3 times with PBS (pH 7.4). Then, the infected cells were fixed with ice-cold 80% acetone (V/V) and stained with FITC-conjugated anti-RABV (Rabies DFA II reagent, Merck, USA) antibody for 1 h at 37°C. 20 fields in each chamber were observed under an inverted fluorescence microscope (Leica Microsystems, Germany), and the 50% endpoint titers were calculated according to the Reed-Muench method [18]. The values were compared with those obtained with the 2^nd^ International Reference Serum (National Institute for Biological Standards and Control, Herts, United Kingdom) and normalized to international units (IU)/ml.

### Statistical analysis

The results of ELISA and RFFIT assays were compared using the linear regression analysis. SPSS 20.0 (IBM, Chicago, USA) was used for Paired t-test analysis and graphs were drawn with the help of Prism 7.0 (GraphPad Software Inc., San Diego, CA) software.

## Results

### Whole virus antigen concentration

The optimum concentration of the purified Vero cell-derived inactivated rabies vaccine (>2.5 IU) antigen which showed a maximum difference between the positive and negative sera was determined (1:400) by checkerboard titration.

### Test sera

The optimal dilution of sera was 1:200 as determined by checkerboard titration.

### ELISA

**Figure-1:**
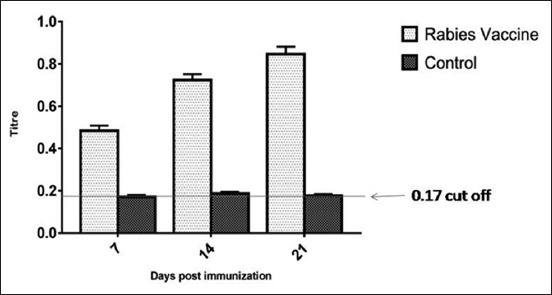
Antibody response in mice determined by enzyme-linked immunosorbent assay. The serum samples from immunized and control group mice were collected and tested for rabies antibody. The mean OD value + 3 standard deviation was considered as the cutoff value, above which the sera samples from the vaccinated mice were taken as positive. Antibody titers of rabies vaccine were significantly different as compared to the control group throughout the study period. Data represent the mean ± standard error. Statistical analysis was done by paired t-test.

The level of the anti-rabies antibody was determined as mean absorbance at 490 nm and was compared to that of the cutoff value (mean OD value + 3 SD). The cutoff value was found to be 0.171, and the serum samples above the cutoff value were considered as positive. As a response to vaccination, all the vaccinated mice showed antigen-specific antibodies in their serum. As per the ELISA results, a significant difference (p<0.005) was observed in the vaccinated groups as compared to unvaccinated control group. Moreover, 7 days post-vaccination onward, a significant increase in the antibody level was observed in the entire vaccinated groups and that increased until 21 days post-vaccination (Figure-1).

### RFFIT

As a response to vaccination, 21 dpi, all the vaccinated mice developed anti-rabies antibodies in their serum. A significant difference was observed in the titers of vaccinated groups as compared to unvaccinated control group. Since the control group has no value or zero value, the cutoff value of this test was not determined. However, it has been observed that an RFFIT titer of 0.5 IU/ml or above gave a good protective immune response in vaccinated animals [19,20].

**Figure-2:**
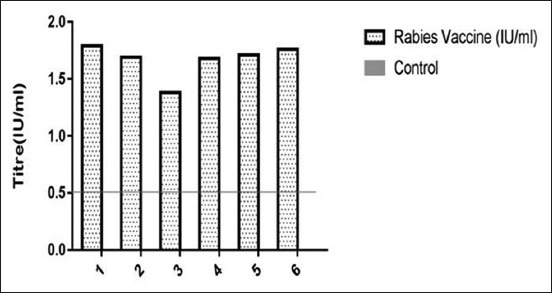
Antibody responses in mice determined by rapid fluorescent focus inhibition test. The serum samples from immunized and control group mice were collected at 21 days post-primary vaccination and tested for rabies antibody. The calculated titer that gave 50% fluorescence in the observed field infected by rabies challenge virus was above 0.5 IU/ml and was considered as the cutoff value, above which the sera samples were considered positive. Antibody titers of rabies vaccine were significantly different as compared to control in the respective mice.

**Figure-3:**
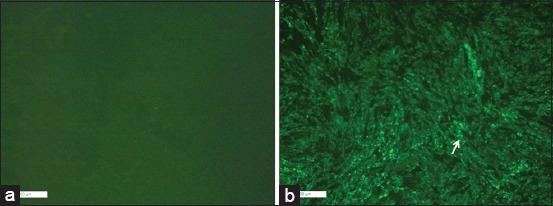
Rapid fluorescent focus inhibition test (RFFIT) in mice. Evaluation of anti-rabies activity of sera using RFFIT in BHK-21 cells using 1:10 dilution FITC tagged (Rabies DFA II reagent, Merck, USA) antibody against rabies virus (a). Cell control with no fluorescence indicates complete neutralization of virus by the tested serum (b). Virus control with specific apple green fluorescence under UV light indicates absence of neutralizing antibodies in the tested sera. Arrow representing the cells infected with rabies virus (apple green fluorescence).

The cutoff value was determined to be 0.5 IU/ml, and the sera samples above the cutoff value were considered as positive from the vaccinated mice (Figures-2 and 3).

### Correlation of ELISA with RFFIT

**Figure-4:**
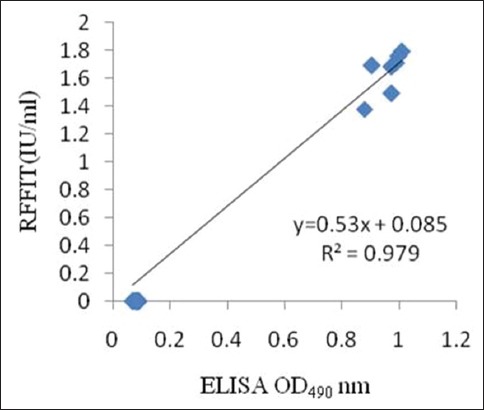
A representative graph showing linear regression between rapid fluorescent focus inhibition test (RFFIT) and enzyme-linked immunosorbent assay (ELISA) on 12 tested sera samples. The linear relationship was obtained between the antibody titer observed in RFFIT and ELISA assay. The linearity was verified (correlation coefficient =0.979; y-intercept =0.085; slope of regression line =0.53).

At 21 dpi, ELISA and RFFIT results were compared (Table-1). The correlation was obtained between RFFIT and ELISA result and linearity was verified by the regression line (R²=0.979) (Figure-4).

**Table-1:** Correlation of ELISA with RFFIT.

Number of mice	Antibody titer	

	ELISA (OD_490_ nm)	RFFIT (IU/ml)
1	1.011	1.79
2	0.905	1.69
3	0.879	1.38
4	0.975	1.68
5	0.990	1.71
6	0.998	1.76
7	0.171	0
8	0.172	0
9	0.170	0
10	0.178	0
11	0.171	0
12	0.161	0

ELISA=Enzyme-linked immunosorbent assay, RFFIT=Rapid fluorescent focus inhibition test

## Discussion

Rabies is a deadly disease to which immune prophylaxis is the only known best way for prevention. The high production cost of vaccine is a major drawback to use vaccines in developing countries. Continuous sero-monitoring following vaccination is one of the major aspects of vaccination strategy. The RFFIT has been considered as the gold standard test for assessing serum viral neutralizing antibodies against rabies virus. The test has many advantages; however the bioassay has different variants such as use of biological materials like BHK-21 cells and virulent rabies challenge virus, which may not be feasible in any virology laboratory, and a greater coefficient of variation limit criterion is required for this viral neutralization assay.

Further, the usage of an international standard reference sample requirement for each sample run makes the assay costly. Thus, in this study, an ELISA protocol was standardized to counteract the disadvantages of RFFIT. ELISA can be performed in any laboratory with an ELISA reader, the sera samples could be monitored at regular intervals, and only a small volume of sera is required as compared to the RFFIT. Further, the assay avoids the usage of virulent challenge virus which requires BSL-3 facilities to handle [21,22]. The short duration of the test with the ability to analyze 50-100 sera samples makes the assay attractive over the RFFIT assay [23]. ELISA has been used as a serological tool to monitor the level of antibodies in wild fox, domestic carnivore, and raccoon [19,24,25].

ELISA was performed with the sera samples from vaccinated and control groups at regular intervals. The antibody levels progressively increased in all the immunized mice during the study period. 21 dpi, RFFIT results indicated an increase in antibody titer in terms of international unit when compared to the standard WHO rabies sera [21]. In RFFIT, an antibody level of 0.50 IU/ml or greater indicated seroconversion following vaccination [10,12,26]. Furthermore, a titer of 0.1 IU/ml corresponding to a complete neutralization at 1:5 serum dilutions in the RFFIT has also been reported [27]. A good correlation study is required for showing any alternative test (ELISA) regarding rabies vaccine seroprofiling in tested animal. In our study, the vaccinated mice had the antibody level of >0.5 IU/ml. For any test to be used as an alternative test to the present one, a correlation study should be required [28-30]. Hence, in this study, at 21 dpi, ELISA and RFFIT results were correlated. Furthermore, the correlation was obtained between RFFIT and ELISA result and linearity was verified by the regression line (R²=0.979).

## Conclusion

Rabies is a fatal disease worldwide caused by rabies virus. In this study, we performed ELISA, as a guiding assay to ascertain the immune status of vaccinated mice before performing RFFIT against rabies virus. In this study, we profiled the serological status of Vero cell-adapted inactivated rabies vaccine through ELISA in mice model that correlated well with the OIE gold standard test RFFIT.

## Authors’ Contributions

AD, SD, and MMC designed the experiment. AD, SD, MMC, DCP, RS, NR, and GM conducted the experiment. AKT, ABP, and VU helped in manuscript preparation. All authors corrected the manuscript, read and approved the final manuscript.
